# Early childhood teachers’ writing beliefs and practices

**DOI:** 10.3389/fpsyg.2023.1236652

**Published:** 2023-11-09

**Authors:** Gary E. Bingham, Hope K. Gerde

**Affiliations:** ^1^Department of Early Childhood and Elementary Education, Georgia State University, Atlanta, GA, United States; ^2^Department of Teaching, Learning, and Culture, Texas A&M University, College Station, TX, United States; ^3^Department of Human Development and Family Studies, Michigan State University, East Lansing. MI, United States

**Keywords:** early writing practices, early writing, teacher beliefs, teacher knowledge, early childhood education

## Abstract

This study examined the early writing beliefs, ideas, and practices of 54 early childhood teachers. Teachers completed a survey designed to examine their early writing beliefs and provided definitions about early writing development through a written response. Teachers were also observed in their classrooms and writing practices were coded for instructional strategy employed by the teacher (i.e., modeling and scaffolding approaches) and the instructional focus of these interactions with attention to early writing skill. Teachers’ definitions of writing often emphasized specific writing skills, with most teachers emphasizing handwriting. Teachers were observed enacting a range of modeling and scaffolding practices to support early writing, but the majority of interactions focused on handwriting supports. Teachers’ definitions of writing and their responses to the teacher belief survey were unrelated to each other, but differentially related to writing skills emphasized in interactions with children. Teachers who identified more than one writing component in their definition were more likely to enact practices to support children’s writing concept knowledge, while teachers who espoused more developmentally appropriate early writing beliefs on the survey were more likely to engage children in spelling focused interactions. Findings have implications for the study of teachers’ beliefs about writing as well as the need for professional learning supports for preschool teachers.

## Introduction

Young children develop substantial early writing knowledge and skills during the preschool years (Diamond et al., 2008; Puranik and Lonigan, 2011; Campbell et al., 2019). This knowledge has led to professional recommendations regarding the types of environmental and instructional supports that early childhood teachers should provide preschool aged children to promote children’s early writing development (Gerde et al., 2012, 2021). However, researchers document wide variability across early childhood settings in the (a) types of environmental writing materials and print resources teachers provide children on a daily basis (Gerde et al., 2015; Zhang et al., 2015) and (b) nature and quality of early writing interactions (Bingham et al., 2017). For example, interactions between teachers and children focused on supporting writing skills are relatively infrequent in comparison to other early literacy and language practices (Pelatti et al., 2015; Zhang et al., 2015). Encouragingly, even though infrequent, studies demonstrate that both environmental and instructional opportunities uniquely contribute to children’s writing development across preschool (Gerde et al., 2015).

Multiple factors have been posited for why early education teachers engage infrequently in early writing interactions and why most instructional interactions in preschool classrooms heavily favor transcription skills (i.e., handwriting and early spelling) rather than early composing (Bingham et al., 2017). For example, researchers have identified that writing practices are related to teacher knowledge about early writing (Bingham et al., 2022), early writing standards (Tortorelli et al., 2021), and pre-service teacher educational experiences (Hall and Grisham-Brown, 2011). Studies linking these constructs and teacher practice suggest that teachers’ social cognitions (their beliefs and attitudes about writing) and their knowledge of how children develop early writing skills likely guide the frequency and types of experiences they provide children. Bingham et al. (2022) illustrate that teachers with more sophisticated early writing knowledge are more likely to provide high quality early writing instructional opportunities designed to support a wide range of early writing skills (i.e., composing, handwriting, and spelling). Because teacher beliefs and knowledge are malleable (i.e., they can be changed), understanding how preschool teachers think about early writing development and how it is promoted in early childhood classrooms is an important area of research.

The purpose of this study was to examine associations among preschool teachers’ early writing beliefs, knowledge, and practices. Because understanding factors associated with teachers’ early writing practices are essential to efforts to support the quality and frequency of early writing opportunities, we were particularly interested in examining how preschool teachers define early writing and their beliefs about developmentally appropriate and inappropriate writing practices. We were also interested in understanding the extent to which teachers’ knowledge and beliefs were related to observed classroom practices. As limited research exists in this area, we explore both qualitative and quantitative approaches for capturing preschool teachers’ beliefs and knowledge.

## Early writing development

Writing is an incredibly complex act, particularly for young children. Even young writers must bring together cognitive, linguistic, motor, self-regulation, and literacy skills into the act of translating thoughts into symbols or marks on a page that have meaning to the child (Dyson, 2001; Berninger, 2009; Chandler et al., 2021). The preschool years, before children enter kindergarten, is a developmental period where considerable knowledge and skill related to writing develops (Puranik and Lonigan, 2011; Rowe and Wilson, 2015), although early marks take many forms that vary in complexity, conventionality, and intention (Rowe and Neitzel, 2010; Quinn and Bingham, 2019). The development of both print and meaning processes for writing emerge as children make connections between what they intend to communicate (i.e., oral language and intention) and the written symbols they generate to communicate these ideas with others (Tolchinsky, 2003; Rowe and Wilson, 2015). Cognitive conceptual models of early writing development typically organize early writing skills into meaning and print or code-based processes (see Kaderavek et al., 2009; Berninger and Chanquoy, 2012) or knowledge strands (see Puranik and Lonigan, 2014). For preschool aged children, these skills are typically broken down into two larger components, namely (a) transcription or procedural knowledge, which contains print awareness, handwriting, and early spelling skills and (b) composing or generative knowledge. The distinction among writing components is included in early learning development standards in preschool and reflects that young children must acquire a variety of skills in early childhood in order to become skilled writers (Tortorelli et al., 2021).

Transcription skills are print and code-based skills required in order to “translate” language into written text (Berninger and Chanquoy, 2012) and include subcomponent skills of (a) print or writing concepts, (b) handwriting, and (c) early spelling (Tortorelli et al., 2021). Writing concept knowledge represents a child’s understanding of how print works (e.g., writing moves in language specific and logical ways from left to right and top to bottom in English and that spaces separate words) and how marks on paper have meaning and can be ‘read’ (Clay, 2000; Rowe, 2008; Puranik and Lonigan, 2014). Writing concepts knowledge, titled conceptual knowledge by Puranik and Lonigan (2014), is complementary to print concepts in that it examines children’s understanding of print within the context of writing. Children’s growing understanding of print is important to their awareness of writing form, which is key to a child’s ability to write conventionally. In preschool, handwriting, or the ability to form letters, emerges as a key developmental indicator of children’s early writing skill, as it represents a complex amalgamation of cognitive, motor, and neuromotor processes (Gerde et al., 2012; Dinehart, 2015). Children’s handwriting reflects their ability to use their fine Dinehart motor skills to manipulate and move a writing utensil, their visual understanding of the letter form, and the knowledge of how English letters are made up of lines and curves (Schickedanz, 1999). Handwriting skills, in turn, support more complex writing skills like invented/estimated spelling as young children develop orthographic knowledge about letters and their formation (Puranik and Apel, 2010; Puranik and Lonigan, 2011).

Increasing sophistication in children’s understanding about letters and letter sound associations support their ability to spell words phonetically (Adoniou, 2014; Sénéchal et al., 2023). Children’s invented spelling abilities begin to develop in predictable ways in preschool and rely heavily on their phonemic awareness skills, particularly their knowledge of letter-sound associations (Puranik and Lonigan, 2014; Zhang et al., 2017). Early spelling development follows a predictable path for English speaking children, with children in preschool and kindergarten moving from pre-phonological to phonological writing (Kemp and Treiman, 2023). As they develop an initial ability to encode sounds in spoken language into text, children first are able to identify and then write the initial sounds in words before moving onto ending and then middle sounds (Ouellette and Sénéchal, 2008; Bear et al., 2012; Cabell et al., 2013). Although 3-year-old preschool aged children, who are mostly likely to be pre-phonological writers (i.e., they are producing some of the symbols of their alphabetic language but not yet using invented spelling; Kemp and Treiman, 2023) are unlikely to write salient sounds in words when asked to write consonant-vowel-consonant words, approximately 30% of 4-year-olds and 50% of 5-year-old-children demonstrated the ability to write either initial or final letters (Puranik and Lonigan, 2011). This is likely one reason that some US states’ preschool writing standards focus attention on letter-sound correspondence and invented spelling skills (Tortorelli et al., 2021). Because spelling reflects a child’s ability to use letters and sounds to encode words (Ehri, 2000), even at the early stages, it taps into orthographic, phonological, and graphophonemic knowledge (Ouellette and Sénéchal, 2017; Kemp and Treiman, 2023).

Composing skills represent children’s ability to generate ideas for what to write and the translation of those ideas into language that is captured in marks on a page (Berninger, 2000; Quinn et al., 2021). Although considerable variation exists in how researchers conceptualize composing skills in early childhood (Quinn and Bingham, 2019), preschool models of early writing and developmental standards designed to guide professional practice often emphasize meaning related skills and processes. Because young children demonstrate composing for varied communicative purposes (i.e., to make a list, label a picture, write a note to a family member, etc.), composing is situated within sociocultural contexts of why one might write and engages both oral language and written language as children attempt to capture their ideas using scribbles, drawings, or letter-like forms (Dyson, 2001; Quinn et al., 2021). Importantly, researchers emphasize that composing is not merely children’s oral response to a writing task, but children’s ability to intentionally connect their oral language to a written product regardless of the writing that is produced (i.e., through drawing, scribbling, or estimated spelling, Rowe and Wilson, 2015; Quinn and Bingham, 2019; Quinn et al., 2021). Approaches for assessing young children’s composing examine the sophistication of children’s ideas, how relevant they are to the writing prompt or context, and how oral responses match or align with written responses that the child shares with an examiner (Rowe and Wilson, 2015; Thomas et al., 2020; Quinn and Bingham, 2022). Consistent across these varied approaches is the importance of thought and communication to writing processes even for young children (Gerde and Bingham, 2023). Because the act of composing involves thought and language in addition to marks on paper, even young children compose before their writing reflects conventionality or properly formed letters (Rowe, 2009; Dyson, 2013).

## Early literacy beliefs and practices

Although there are few studies examining preschool teachers’ beliefs about early writing specifically, researchers have studied early childhood literacy focused beliefs, which sometimes contains attention to writing. Across studies, early childhood teacher beliefs have been conceptualized and measured in varied ways (Charlesworth et al., 1993; File and Gullo, 2002; Hindman and Wasik, 2008), but inherent across conceptualizations is that beliefs reflect ideas that are valued by an individual and perceived as factual or true (Evans et al., 2004). Conceptualizations of teachers’ beliefs often emphasize teachers’ thoughts and assumptions about (a) an area of development [e.g., knowledge or ideas about early literacy development, and/or (b) the importance of certain pedagogical approaches for supporting that development (i.e., the belief that there are best ways to support children’s learning)]. Inherent in the study of beliefs is the importance of teacher knowledge, as research suggests associations among these constructs (Hindman and Wasik, 2008; Schachter et al., 2016). Because researchers have approached the study of beliefs with such varied conceptualizations, they have used a number of qualitative and quantitative approaches for understanding how teachers think about early literacy broadly (Cunningham et al., 2009; Campbell et al., 2019). In other words, because beliefs represent teachers’ understanding about development and their perceptions about how certain practices support that development, it is important to attend to teachers’ literacy beliefs within the context of instructional practices.

It is long known that teachers’ beliefs inform their pedagogical decision-making and are a filter by which teachers perceive the importance of certain instructional approaches (Richardson et al., 1991; Fenstermacher, 1994). Cunningham et al. (2009) found that teachers’ early literacy beliefs related to how they allocated time for literacy instruction.

Others document that teachers’ beliefs relate to specific literacy practices they enact in the classroom (e.g., Stipek and Byler, 1997; Scull et al., 2012). For example, in their survey of Head Start teachers, Hindman and Wasik (2008) found that teachers’ early literacy beliefs varied somewhat by the early literacy skill being assessed. Although teachers were much more likely to endorse the importance of certain instructional experiences for supporting language skills, they tended to not endorse active teaching of code-based skills, a finding replicated by Schachter et al. (2016). In their study of teachers and parents’ beliefs about reading, Evans et al. (2004) found that teachers who endorsed graphophonemic views of reading were more likely to rate phonics and letter sound instructional activities as important, while teachers endorsing constructivist views of reading that emphasize language and meaning processes were more likely to endorse the importance of contextual approaches, such as using books with natural language, for supporting children’s reading development. Similarly, in a study by Campbell et al. (2019), teachers’ endorsing child-centered and play-based literacy beliefs reported engaging children in play-based literacy interactions and were more likely to resist commercially developed phonics programs.

However, others have found limited associations among early childhood teachers’ beliefs and practices (Hamre et al., 2012; Sandvik et al., 2014) or even negative associations (Schachter et al., 2016). For example, in their study of Norwegian teachers, Sandvik et al. (2014) found that preschool teachers held early literacy beliefs that were generally aligned with current research on children’s early literacy development, but that their self-reported literacy practices did not reflect such beliefs. Others have noted discrepancies among beliefs and practices when beliefs are self-reported and classroom practices are examined via observations (McMullen et al., 2006). Schachter et al. (2016) found limited relations between teachers’ beliefs of some literacy skills and practices (e.g., beliefs about book reading and book reading instructional practices) and negative associations among beliefs and practices for other literacy skills. The negative associations were noted between (a) teachers’ code-based beliefs and observed code focused instruction and (b) teachers’ oral language and vocabulary beliefs and classroom instruction designed to support these skills. In their discussion of their findings, they raise concerns about the fact that many survey based measures designed to assess teachers’ beliefs may be impacted by social desirability because teachers understand how to answer such questions. An additional explanation for weak or unexpected associations between beliefs and practices may result from the fact that many preschool classrooms offer children’ literacy focused interactions that are of low quality (Justice et al., 2008; Schachter et al., 2016). Adequate levels of both beliefs and instructional practice may be needed in order to find an association among constructs.

## Early writing beliefs and practices

In contrast to early literacy beliefs, we know very little about how preschool teachers view writing, how they define it, and which practices they believe promote young children’s writing development. Early educators’ beliefs about writing may function differently from their beliefs about literacy broadly for various reasons. First, early childhood teachers receive limited, if any, pre-service teacher education coursework focused on early writing pedagogy (Zimmerman et al., 2014). Moreover, preschool teachers enter the profession from a range of backgrounds with varied educational training and experiences (Maxwell et al., 2006; Whitebook et al., 2009); only some of them from traditional teacher education programs. Limited educational experiences learning about writing development and pedagogy may be why teachers report relying on their own K-12 schooling experiences to inform their ideas about teaching writing (Ng et al., 2010). Unfortunately, these experience-informed beliefs are often negative or emphasize handwriting and spelling rather than composing and/or purposes for writing (Colby and Stapleton, 2006; Mackenzie, 2014). The negative writing experiences that teachers reported they had as students themselves (Colby and Stapleton, 2006; Hall and Grisham-Brown, 2011) may be why early educators, at least in the US, limit their writing time and opportunities in the classroom or focus on a narrow set of writing skills (Pelatti et al., 2015; Zhang et al., 2015; Bingham et al., 2017). A second reason relates to the fact that early childhood teachers may not consider certain writing experiences as developmentally appropriate for young children or that children may not benefit from writing instruction unless they are interested in writing. This may be one reason that some teachers endorse a “readiness perspective” for how and when they might provide writing instructional experiences to young learners (Gerde et al., 2019a).

In contrast to this perspective, research also identifies that teachers believe that young children find writing to be interesting (Gerde et al., 2019b) and that they identify young children as writers early on (Hall et al., 2019; Magnusson et al., 2022). For example, Hall et al. (2019) found that early educators had more positive beliefs about preschoolers’ writing abilities than parents, demonstrating an understanding of writing development that was not typical of other adults. Survey research using researcher-generated items from the Preschool Teacher Literacy Belief Questionnaire (TBQ; Seefeldt, 2004) identifies that preschool teachers vary considerably in their writing development and instructional beliefs (Hindman and Wasik, 2008; Schachter et al., 2016). Whereas most teachers tended to espouse beliefs indicating their understanding that scribbling and drawing are important to young children’s writing development and that children should write without worrying about spelling, other teachers disagreed with such statements and also the perspective that children learn writing skills through teachers’ modeling how to write. Unfortunately, Schachter et al. (2016) were unable to link teachers’ beliefs on the TBQ to observed instructional practices because so few teachers were observed engaging in writing interactions with children. Findings from these studies suggest the need to examine beliefs in a holistic fashion as preschool teachers may hold varying beliefs that may not be reflected adequately in researcher-generated categories. In addition, research that focuses on a wider range of early writing practices that have been shown to be predictive of children’s early writing development (see Gerde et al., 2015) is needed.

Qualitative research coding open-ended responses about teachers’ beliefs of early writing identified three teacher views on young children’s writing (Gerde et al., 2019b). One group of teachers held an affirmative belief that young children enjoy writing. Other teachers held a conditional belief that some children do, and some children do not, enjoy writing depending on the child’s characteristics. For example, teachers believed that (a) boys compared to girls, (c) younger children vs. older children, or (c) children with less developed fine motor skills tended to not enjoy writing. Finally, a third group of teachers held a belief that children enjoyed writing when teachers created learning experiences that made writing fun, primarily through varied and interesting materials (e.g., whiteboards, scented markers). Only six of the 32 teachers from their study discussed creating meaningful writing opportunities for children to compose; and, interestingly, these teachers represented all three belief categories. In other words, teachers can hold varied and somewhat conflicting beliefs about children’s writing development. No pattern emerged identifying a relation between these belief categories and teachers’ practices. Moreover, teachers’ educational background, teaching experience, curriculum, and program type (e.g., Head Start, state funded) did not predict their beliefs.

There are likely multiple factors that influence teachers’ ideas about writing, including limited and varied opportunities to learn about writing development and pedagogy (Zimmerman et al., 2014) and potentially negative experiences with writing as they learned this important communication skill (Colby and Stapleton, 2006). In addition, there is extensive complexity in early writing development, which may contribute to ideas that some children need particular skills (i.e., fine motor) before they are “ready” to write (Gerde et al., 2019a). This may be the case for teachers who are less knowledgeable about how children’s marks on the page can provide important information into their writing concept, transcription, and composing skills (Bingham et al., 2022). The complexity of early writing development for young children may be taken for granted by adults who have long automated developmental systems that take years to fully develop, which may lead to developmentally inappropriate writing instruction (Puranik and Lonigan, 2014; Bingham et al., 2017). While initial work in the US examining early educators’ beliefs and writing practices identified limited relation between beliefs and reported or observed practices (Gerde et al., 2019b), in a study of pre-service preschool educators from Norway, Sweden, and Finland, Magnusson et al. (2022) found that preschool teachers endorsed play based approaches for supporting children’s writing, which was also reflected in their self-reported practices, particularly when discussing ways to make writing environments interesting and engaging for children. However, they also found that teacher’s self-reported writing mediation practices lacked details and concrete examples. Given such findings, it is clear that we need to continue to investigate teachers’ beliefs about early writing in ways that appreciate the complexities that are influencing teachers’ beliefs and the complex nature of writing development. The use of both theory-informed researcher-developed categories and qualitatively teacher-derived ideas may be essential for understanding teachers’ complex beliefs and how they relate to the decisions they make about designing and supporting writing opportunities in early childhood classrooms.

## Current study

The current study was designed to examine teachers’ beliefs about children’s writing development through both qualitative and quantitative means and to determine the extent to which these beliefs are related to their observed instructional practices. Because there are few measures designed to expressly examine early childhood teachers’ early writing beliefs, we used a previously validated scale (i.e., the TBQ) along with an open-ended question designed to elicit teachers’ ideas about children’s writing development. Three research questions guided this study.

### Research questions

1. How do teachers define early writing development? Given previous research studies examining teachers’ early writing beliefs and knowledge (Gerde et al., 2019a; Bingham et al., 2022), we hypothesize that teachers will define early writing in various ways that describe transcription related skills (writing concepts, handwriting, early spelling) while focusing less on composing related skills.
2. How are these definitions related to self-reported writing beliefs as assessed through the TBQ? Because previous research suggesting that teachers’ beliefs and knowledge are related (Hindman and Wasik, 2008), we hypothesize that teachers’ beliefs as measured by the TBQ will be positively related to the number of components they articulate in their definitions.

To what extent are teachers’ definitions of writing and writing beliefs as assessed by the TBQ related to their writing practices? As previous research documents some associations between teachers’ literacy beliefs and their practices (Schachter et al., 2016), we anticipate that teachers’ writing beliefs and definitions will be positively related to their early writing practices.

## Materials and methods

### Participants

A total of 54 lead Head Start teachers from two US states participated in this study. Teachers provided instruction to preschool aged children (ages 3 to 5 years old) in mixed aged classrooms. The majority of teachers in the sample reported their race as Black (57%) with the remaining participants identifying as White (43%). Teachers were relatively experienced, reporting that they had been teaching preschool children for an average of 8 years (*SD* = 7 years, Range = 6 months to 30 years). Consistent with Head Start requirements, the majority of teachers in this sample reported having a Bachelor’s degree (61%), or Master’s degree (28%), while the remaining 11% reporting having obtained an Associate’s degree. The majority of teachers were teaching in programs using the Creative Curriculum (80%).

### Procedures

Teachers in this study were participating in a professional development (iWRITE; Gerde and Bingham, 2023) project aimed at supporting their early writing practices. Data are taken from the first time point of the study, with information collected in the months of September and October, before any professional development was experienced. We recruited early childhood programs from two US states (one Southern and one Midwestern), with approval to engage in the study being granted by early childhood program directors. Once approval was obtained, researchers visited programs to discuss the study with teachers and invite participation in the study. Teachers were provided information about the study and an opportunity to ask questions from the researcher before they were asked to sign a consent form if they were interested in participating. Participants who agreed to participate in the project were asked to (a) complete a demographic survey about themselves and their educational and work experience, (b) complete a survey that contained both open-ended and Likert items designed to assess their early writing beliefs, and (3) participate in an observation of their classroom practices.

Classroom observations, which included videotaping of instructional practices, occurred during weeks six to tenth of instruction of the school year (i.e., months of September and October) during a typical day of instruction. Observations typically lasted approximately a full morning of instruction (approximately 2 h of indoor learning, excluding outside play) so that researchers could document the literacy practices that teachers typically enacted on a daily basis. At both the beginning and the end of the classroom visit, observers confirmed with the teacher that the observed instruction represented a typical instructional day. Video recording of preschool teachers’ instructional practices focused on any instructional routines where writing might be present, including: breakfast or snack time, large group or morning meeting time, shared book reading, centers or free choice activities, and, if offered by the teacher, small group instruction. Videos were uploaded into a video editing program (INTERACT) and coded for a variety of modeling and scaffolding strategies (see section “Measures and coding”).

### Measures and coding

Early writing beliefs were assessed through a survey that teachers completed before they were observed in their classrooms. Teachers responded to an open-ended prompt asking them to define early writing development and also responded to a series of statements about children’s early literacy development. We briefly describe each approach.

#### Definition of early writing development

Given previous research suggesting the importance of teacher knowledge to their early writing beliefs and practices (Hindman and Wasik, 2008; Magnusson et al., 2022) teachers were asked to define early writing development. This open-ended response took up approximately a half-page of the survey at the beginning, allowing teachers ample space to write their responses. Teachers’ open-ended responses were entered verbatim into an Excel spreadsheet and a second coder double checked them for accuracy. The spreadsheet was then uploaded into Dedoose, an online data management and coding platform,^1^ for coding. We used a two-step process to analyze teacher’s definitions. First, we used an *a priori* set of codes, derived from well-established theories of early writing (Kaderavek et al., 2009; Puranik and Lonigan, 2014; Kim, 2020), to identify language reflecting writing components: writing concepts, handwriting, spelling, and composing. Second, two coders independently reviewed definitions using a descriptive coding process (see Saldaña, 2015) in order to identify key writing beliefs identified by the participants that were not originally included in our *a priori* coding. This resulted in additional codes, such as “developmental progression of writing skills” (explained below), that were then included in the codebook. Once the code book was finalized through this two-step process, teachers’ definitions were then evaluated by two PhD level graduate students with expertise in early literacy development and previous experience as early childhood educators. Responses were double coded by these research assistants revealing strong agreement across writing samples (0.91). Disagreements were discussed with the two authors of this study and final coding was agreed upon by all scorers.

#### Preschool teacher literacy beliefs questionnaire

Teachers completed the Preschool Teacher Literacy Beliefs Questionnaire (TBQ; Seefeldt, 2004; Hindman and Wasik, 2008), which contains 24 items designed to assess early childhood teachers’ literacy beliefs. Items on the TBQ focus on 4 early literacy domains, namely (1) oral language/vocabulary, (2) book reading, (3) code-related skills, and (4) early writing. Items ask teachers to consider both skills that young children should be developing and specific instructional practices for how teachers should support these skills. Teachers are asked to rate their agreement with statements on a five-point Likert scale (1 = strongly disagree to 5 = strongly agree), with some items worded negatively and then reverse coded. Teachers with higher scores on the TBQ are considered to have beliefs that are more closely aligned with research-based notions of how children learn language, reading, and writing skills. We were primarily interested in writing subscale of this questionnaire, which is made up of six items designed to assess teachers’ beliefs about early writing development (e.g., “Should write without worrying about spelling” and “Children learn to read before learning to write,” reverse scored), how children learn to write (i.e., “Children learn to write by watching teachers write”), and classroom practices designed to support early writing (e.g., “Should not write until teachers show them how to form each letter,” reverse coded). Survey responses evidenced acceptable levels of internal consistency for the total scale (α = 0.68) and the writing subscale (α = 0.62). These alphas are lower than reported by Hindman and Wasik (2008), but higher than those reported by Schachter et al. (2016). Scores on individual items were summed to obtain summative ratings of teachers’ beliefs about children’s writing.

#### Early literacy practices

Video coding of teachers’ observational data examined early writing pedagogical supports available to children using the measure Writing Resources and Interactions in Teaching Environments (WRITE; Gerde et al., 2015). Using an expanded coding structure outlined in previous work (Bingham et al., 2017, 2022), we examined videos in order to identify (a) the instructional focus of interactions (i.e., handwriting, spelling, composing, writing concepts), and (b) the teaching strategy that teachers were using to support children’s writing (e.g., modeling and scaffolding interactions). Modeling interactions included teachers’ practices aimed at demonstrating purposes of writing (“I am going to number the things we need at the store as I make my list for our class party.”) and explicit directions or demonstration of writing concepts (“I am going to draw a ‘T’ by making one line down and one line across”). Scaffolding interactions were focused on how teachers (a) broke down writing tasks to make the task easier for children (e.g., stretching sounds in words to support children’s spelling or supporting children’s idea generation to focus their thinking on something that they might write) and (b) expanded children’s involvement or thinking about writing in a manner that pushed thinking or skill development (e.g., encouraging children to compare the ideas generated by multiple children to reach a consensus for a book title, encouraging children to analyze and compare various letter forms). Previous work with the original and expanded WRITE indicate that the measure has good internal consistency (α = 0.76) and construct validity as evidenced by its correlation to the Early Language and Literacy Classroom Observation scale (ELLCO; Smith et al., 2008) (*r* = 0.66, Gerde et al., 2015). Scores from the Writing Interaction scale on the WRITE have been shown to relate to children’s writing development, indicating that the measure has good predictive validity (Gerde et al., 2015; Bingham et al., 2017).

#### Coding of writing practices

Videos were coded by five early childhood literacy experts who were former early childhood teachers and who had received or were receiving a PhD in early childhood education. Two coders independently identified writing events in videos and time stamped them to ensure that we captured all instances of teacher-child writing. Coders were trained to examine each teacher-child writing interaction or utterance (i.e., what teachers said during interactions) for evidence of writing component focus (writing concepts, handwriting, spelling, and composing) and instructional strategy (modeling, scaffolding to make the task easier, scaffolding to expand child’s involvement or understanding). As part training, coders familiarized themselves with the codes, definitions, and examples from previous research and coded several videos in order to establish baseline interrater agreement with the second author. Once all coders reached 90% interrater agreement with master codes, they were split into teams of two randomly and they coded all writing interactions for writing component and strategy. This ensured that all teacher practice data was double coded. When disagreements emerged between coders, these were resolved through conversations and the agreed upon codes were used in analyses. There was high agreement between coders before resolving disagreements for both writing component (93%) and writing strategy (86%).

## Results

To answer research question 1, *how do teachers define early writing development*, we examined the qualitatively coded data to identify the frequency of each component teachers mentioned in their responses and other ideas teachers generated. Considerable variability existed in teachers’ definitions of writing, as teachers emphasized different component processes (writing concepts, handwriting, spelling, and composing) as well as the developmental progression inherent in young children’s early writing development. Representative statements of teachers’ responses are displayed in Table 1 along with the total number of teachers representing each code. Because teachers could have discussed multiple component skills in their answers, categories in Table 1 are not mutually exclusive (i.e., responses total to more than the number of teachers in the sample). Although definitions generally aligned with research-based conceptualizations or US preschool early learning standards, teachers heavily emphasized some component processes, such as handwriting (85%) and writing concepts (31%) significantly more than others (i.e., spelling, 15%, composing, 20%). Patterns in teachers’ responses are discussed below.

**TABLE 1:** Preschool teachers definitions of early writing by writing component.

Writing concepts	Handwriting	Spelling	Composing
***N* = 17**	***N* = 46**	***N* = 8**	***N* = 11**
Early writing development is in children’s scribbles, drawings and writings. When children are exposed to literacy and print, they begin to understand writing carries meaning.	Early writing development help children with their small motor skills. “It also helps children form some letters and shapes.”	“Letter and letter sound recognition. Scribbles to mock letter forms.”	I would define early writing development when a child begins to scribble and tries to draw objects and lines to communicate ideas.

Teachers could indicate more than one component in their written responses.

Forty-six teachers (85%) emphasized handwriting skills in their definitions of early writing. Teachers discussing handwriting in their answers often positioned writing as “Children learning how to form letters…” or “the form it takes” to generate writing. Overwhelming, teacher responses that discussed handwriting skills also emphasized fine motor skills. This is illustrated by one teacher who suggested “I define early writing development as any form of fine motor hand (using any form of writing utensil) movement expressed on paper, or any other surface.” Similarly, another teacher suggested “Early writing development helps children with their small motor skills. It also helps children form some letters and shapes.” In some responses emphasizing handwriting skills, teachers also discussed a developmental progression of skills as children’s movements become more coordinated. Consider the following quote “Any marks children make to represent writing using drawing and writing tools. Eventually these marks will start to form shape like letters, and then they begin to form letters.” In this last example, the teacher also emphasized conceptual knowledge or writing concepts, the second largest category emerging in teachers’ definitions.

Seventeen teachers (31%) discussed children’s conceptual knowledge or understanding of writing concepts. These responses primarily articulated the connection between oral and written language. As one teacher noted, “Early writing development is when children use symbols to make connections between spoken and written language.” Another teacher emphasized writing concept knowledge by suggesting that writing was “Any purposeful marks or exploratory marks made by a child.” A few teachers’ responses that were categorized as emphasizing writing concepts articulated how children use different writing tools (i.e., “Any marks that children make to represent writing using drawing and writing tools”). Although no teachers emphasized writing concept knowledge related to linearity or directionality (e.g., writing from left to write), teachers including writing concepts in their definition did sometimes discuss print explicitly (i.e., “The exploration of print and its uses, the form it takes, and its meanings.”). Only one teacher mentioned punctuation in their response, suggesting that it was too early to focus on in preschool, “Punctuation used improperly at first grade with a gradual proper use of!,.,?”

Only eleven teachers (20%) in this sample mentioned composing related concepts in their definitions of writing. Teachers who articulated composing in their definitions emphasized the importance of communicating thoughts or ideas, such as, “Early writing development is when children are beginning to understand that writing is how we communicate. In Head Start, students sometimes communicate by drawing and telling adults their story of their pictures.” Teachers who tended to emphasize composing skills were also likely to mention other early writing skills in their responses, particularly writing concepts or the ability to link spoken and written language in intentional ways. As one teacher emphasized, writing is “…putting something down on paper and being able to articulate what it is.” Rarely (4% of responses) did teachers who discussed composing skills specifically talk about how discussing ideas before or during writing or brainstorming. In one rare exception, a teacher suggested “Early writing development are also the pre writing like brainstorming letter and word formations children do even before being presented paper or pencil.”

Teachers’ definitions focused the least on children’s early spelling development. Only eight teachers (15%) articulated how writing including children’s ability to hear the sounds in spoken language. When teachers talked about early skills that support children’s early spelling development, responses primarily emphasized symbol and sound relationships, particularly how letters make sounds that children must learn to be able to write. For example, one teacher suggested that writing involves “…understanding symbols, sounds, and language” while another suggested that writing is about “learning to form letters and sounding them out.” Only one teacher used the term “invented spelling” and she did so when describing the developmental progression of writing skills (e.g., “writing will progress from letter strings to then invented spelling”).

As briefly mentioned earlier when discussing handwriting skills, 31% of responses explicitly mentioned an early writing developmental progression, or stages, that children follow as they develop early writing skills. Teachers in this group tended to emphasize that children moved from less sophisticated to more conventional writing, noting that children’s early writing contains scribbles or drawing before they learn to write letters. As one teacher noted, children “…scribble, make letter-like forms, trace letters, and write letters.” Teachers’ descriptions of early writing progressions overwhelmingly focused on the form of children’s writing, and frequently made reference to handwriting skills. This is reflected in the following definition “children are beginning to learn pencil control and scribbling and mock like letters.” Other developmental progressions noted that both writing progress and a developmental progression that included spelling skills. As one teacher articulated, “It starts the first time they pick up a tool they can make marks with. Eventually, the marks become meaningful to them. Then they go through stages of advancement as they learn letters and sounds.” Teachers that discussed early writing as a developmental progression often positioned preschool children’s writing as involving distinct phases and they provided examples of this progression (e.g., “They have different levels of early learning, some begin with lines, go onto forming some letters, then progress to making real letters.”). A common thread through teacher definitions that noted the developmental nature of young children’s writing skills was that early writing was the beginning of a process (e.g., “Early writing development is the beginning to these kids writing”).

A sizable percentage of teachers (37%) also included how they would support early writing skills in their definition. For example, one teacher who emphasized handwriting skills suggested, “Early writing development would be the practice of introducing students to writing practice exposure to different tools: pencil, markers, crayons, paper. Also, it would be the practice of getting their hands and arms ahead (dexterity to write in the perfect manner).” As evident in this example, recommendations that teachers included in their definition overwhelmingly focused on handwriting skills in addition to exposure to various writing tools and opportunities to strengthen fine motor skills (e.g., “Experiences that allows children to practice fine motor skills that later help with writing.”). A much smaller group of teachers who emphasized writing related activities in their definitions, discussed the importance of varied experiences with print and books (e.g., “Give as much exposure to all kinds of print – stories, modeled writing, environmental print, etc. Oral language development is also crucial before we can expect to see a lot of written literacy.”).

Before we address research question 2, *How are these definitions related to self-reported writing beliefs as assessed through the TBQ?*, we first want to draw attention to the teachers’ scores on this assessment. As displayed in the means of Table 2, teachers’ scores on the writing subscale of the TBQ fell, on average, between “neither agreeing or disagreeing” (3) or agreeing (4). This suggests that teachers tended to positively endorse items on the scale, but did not hold the beliefs strongly. To examine research question 2, we generated a non-parametric test using the Wilcoxon signed rank sum test. We used this data analytic approach because teachers could have supplied 0, 1, 2, 3, or 4 writing components in their writing definitions. Given that few (*n* = 5) teachers emphasized three components (and none identified four writing components) in their definitions, we combined categories 2 and 3 to create a group where teachers emphasized multiple writing components. As a non-parametric test, the Wilcoxon signed rank test allowed us to determine whether teachers in these three groups differed in their writing beliefs as measured by the TBQ. Hence, our analyses compared the extent to which teachers in three groups (i.e., 0 writing components, 1 writing component, and 2 or more writing components) held similar beliefs about children’s writing development and how it is promoted. Our findings revealed a non-significant test, suggesting that teachers’ definitions of writing were independent from their self-ratings on the TBQ.

**TABLE 2:** Correlations, means, and standard deviations of study variables.

	1	2	3	4	5	6	7	8	9	10
1. Early writing beliefs (TBQ)	–									
2. Teacher education	0.18	–								
3. Teacher experience	−0.30*	0.09	–							
**Writing interaction focus**										
4. Writing concepts	0.06	0.17	0.01	–	–					
5. Handwriting	−0.05	−0.08	0.05	−0.05	–					
6. Spelling	0.34*	−0.03	−0.02	−0.10	0.28*	–				
7. Composing	−0.26	0.23	0.17	0.11	−0.26	−0.06	–			
**Writing strategies**										
Modeling Scaffolding-Making	0.10	0.07	0.01	0.33*	−0.06	0.07	0.36**	–		
9. Scaffolding-making writing easier	0.14	0.01	0.01	−0.05	0.36 **	0.52**	0.20	0.54**	–	
10. Scaffolding expansions	0.21	0.01	−0.02	0.04	0.15	0.57**	0.40**	0.63 **	0.70**	–
Mean	20.10	2.17	9.39	4.31	13.94	8.52	6.72	6.78	16.63	8.98
*SD*	2.87	0.61	7.14	6.53	20.69	12.04	10.98	7.80	18.40	12.85

**p < 0.01, *p < 0.05.

To examine research question 3, *To what extent are teachers’ definitions of writing and writing beliefs as assessed by the TBQ related to their writing practices?*, we explored possible associations between the number of writing components teachers named in their writing definitions and their instructional practices coded as emphasizing writing concepts, handwriting, spelling, and composing. To analyze these relations, we generated a number of non-parametric tests using the Wilcoxon signed rank sum test. Similar to our approach in addressing research question 2, we used the combined categories of 2 and 3 to create a group where teachers emphasized multiple writing components. We then compared the extent to which teachers in these groups were enacting similar writing practices. Results demonstrate that teachers’ writing definitions were generally unrelated to their observed instructional practices. The one exception to this pattern was that teachers emphasizing multiple writing components in their definitions were more likely to be observed enacting writing practices that emphasized writing concept knowledge (*W* = 22; *p* < 0.05). In other words, teachers who defined writing as involving multiple writing skills were more likely to be observed emphasizing the relation between oral and written language in their instructional practices and drawing attention to features of how English print works (left to right, with spaces, placement on a page) than teachers who did not emphasize separate writing components in their definitions.

We also examined the extent to which teachers’ writing beliefs as measured by the TBQ were related to (a) the writing component (writing concepts, handwriting, spelling, and composing) they emphasized in observed interactions with children and (b) the instructional strategy (modeling, scaffolding by making the task easier, scaffolding by expanding). We display these associations in Table 2. As evident by this analysis, teachers’ beliefs on the TBQ were largely unrelated to their observed writing practices. One exception to this pattern of null associations is a positive relationship between the TBQ and the number of spelling related writing interactions between teachers and children. This association is likely a function of the fact that some TBQ items emphasize spelling related skills and their association with early writing skills. Interestingly, teachers’ beliefs about writing were negatively related to their years teaching preschool aged children, but unrelated to their educational backgrounds.

## Discussion

We designed this study to examine teachers’ beliefs and ideas about early writing and their association with observed classroom practices. As one of the few studies to date that examines these constructs, the current study employed both quantitative and qualitative methods to describe preschool teachers’ understanding of early writing development and explore how they were related to the instructional focus of teacher-child writing interactions. Because beliefs are argued to have a knowledge-based component (Hindman and Wasik, 2008), and recent research demonstrates how preschool teachers’ writing knowledge facilitates early writing practices (Bingham et al., 2022), we were interested in examining associations between how teachers defined early writing and their beliefs about early writing development. Our findings point to limited concordance between teachers’ beliefs as assessed by a previously validated measure of early writing and writing definitions, but each was associated in differential ways to the instructional focus of teacher-child writing interactions. We discuss main findings, recommendations for future research, and implications for professional practice below.

### Variability of teachers’ beliefs and ideas about writing

Consistent with our hypothesis, teachers participating in this study reported a wide variety of beliefs and ideas about early writing development in both their qualitative self-derived responses and their quantitative responses to researcher-generated items. Across both response options, teachers generally endorsed developmentally appropriate belief statements as measured by TBQ and also defined early writing skills to include a number of components that align with research-based notions of early writing that are articulated in US preschool writing standards (Tortorelli et al., 2021). It is noteworthy that teachers’ definitions primarily focused on handwriting and print concept skills, with less attention to composing skills and early spelling skills, such as invented spelling. In many definitions that emphasized handwriting as a key writing component, teachers highlighted the importance of children’s fine motor development and their ability to form letters for writing development. Consistent with both teacher belief (Gerde et al., 2019b) and early writing skill research (Chandler et al., 2021), it was clear that for many teachers writing was synonymous with handwriting and that developing strong fine motor skills was prerequisite for successful writing.

Teachers also showed some understanding about developmental progressions of writing, but many of these statements focused almost exclusively on form or development of fine motor skills as being the end point of writing in preschool rather than emphasizing early spelling skills, such as invented spelling. Rarely did teachers articulate ideas about making connections between letters and sounds or using letters to build words. Although it could be argued that focusing on spelling skills in preschool is inappropriate for young children, it is important to acknowledge that early, or pre-phonological, spelling development begins with children’s understanding of, and ability to use, letters in their writing (Kemp and Treiman, 2023). As studies document that many children in US preschools are able to write letters and even engage in invented spelling (Puranik and Lonigan, 2011; Guo et al., 2018), it is important that teachers are engaging children with opportunities to use and connect early reading (decoding) and writing (encoding) skills (Cabell et al., 2013). Because phonemic awareness is at the core of children’s ability to segment the sounds within words to be spelled (Zhang et al., 2017; Sénéchal et al., 2023), teachers understanding of early spelling skills and their ability to engage students in a manner that supports their ability to hear the sounds in spoken language at the syllable, onsets and rimes, and phoneme level are key to supporting reading and writing development (Hall et al., 2015; Piasta, 2023).

In written responses, sophisticated or detailed descriptions of writing development that focused on composing processes or how children can connect oral language to written language was rare. It was uncommon for teachers to articulate that early writing included learning about the purposes for writing and developing skills for generating ideas, selecting words to use in their messages, or making connections between oral and written language. This finding may be a limitation of having participants write their responses or may reflect a more constrained understanding about children’s writing, something noted in the literature (Bingham et al., 2022). In their study examining teachers’ knowledge and practices, Bingham et al. (2022) found that teachers demonstrated a strong understanding of children’s handwriting development and the importance of being able to write letters, but showed a more limited understanding about how drawing and writing involved communicative processes. However, as we will discuss in detail later, it is teachers who have a more complex and thus, more complete understanding of the multiple components of early writing who provided more practices supporting conceptual knowledge of early writing, practices we know support children’s early writing development (Bingham et al., 2017).

A possible reason that preschool teachers appear less likely to discuss composing related skills when defining children’s writing may be a result of the overwhelming focus on handwriting skills in US preschool early learning standards (Tortorelli et al., 2021). Standards have been known to inform teachers’ practices for a range of skills (Scott-Little et al., 2012), but may unintentionally narrow teachers’ beliefs about the importance of skills not contained in standards. Other explanations may be related to teachers’ educational experiences and early childhood curriculum. Early childhood educators have multiple pathways to the profession that results in highly varied educational backgrounds (Maxwell et al., 2006; Whitebook et al., 2009). Even those teachers with an associate or bachelor’s degree in education or child development may not have had courses or even course content in early writing development or practicum experiences to support early writing skills (Zimmerman et al., 2014). Teachers with minimal formal educational experiences about early writing may turn to curricula for guidance. However, even the most widely used early childhood curricula in the US provide uneven resources for early writing that do not reflect the full conceptual model of early writing to include composing, spelling, and handwriting or provide minimal guidance for supporting writing in ways that promote children’s early development (Gerde et al., 2019a). Given that teachers’ education was not related to teachers’ beliefs and that years of experience was negatively related to beliefs, it appears that stronger pre-service and in-service teacher learning opportunities are needed that focus on supporting teachers’ developmentally appropriate writing beliefs, knowledge, and skills.

Teachers primary focus on handwriting skills in their responses may provide insight into why writing opportunities are so rare in preschool classrooms (Gerde et al., 2015) or of so low quality (Bingham et al., 2017). For example, for literacy broadly we know teachers’ beliefs inform their practices (Bingham and Kenyon-Hall, 2013; Schachter et al., 2016). For teachers who consider writing to be primarily handwriting, they may consider writing opportunities that go beyond writing one’s name or tracing to be developmentally inappropriate for young children. Rather, as we see in this study, they may perceive developmentally appropriate writing opportunities to focus on the development of strong fine motor skills in preschool so that children will be “ready” for the writing expectations in kindergarten and later grades. Perhaps this is why we observe ample opportunities for children to write their name with a range of materials, opportunities for tracing and copying letters, and experiences for exercising fine motor skills available at writing centers (Gerde et al., 2015). This readiness perspective is not unusual among early childhood educators and may be how those beliefs and ideas are manifested for early writing through the provision of writing materials and activities (Gerde et al., 2019b; Magnusson et al., 2022).

### Different approaches for eliciting beliefs and ideas offer unique insights

According to the responses teachers provided in this study their definitions of early writing and beliefs about early writing were unrelated, suggesting that they were tapping into different understandings about early writing development. This finding was opposite of our hypothesis that beliefs and writing definitions would be related. Results may reflect our elicitation approach of gathering teachers’ definitions by having them write their own ideas, which some teachers may have found challenging. However, this approach also allowed teachers to share their understanding about early writing without limiting responses to preconceived categories. A primary focus in teachers’ written definitions that resulted in a heavy emphasis on handwriting skills, while attending less to other writing skills, may have made it challenging to find an association with the TBQ. Alternatively, the fact that teachers’ responses on the TBQ evidenced only acceptable reliability may have also contributed. In their original study, Hindman and Wasik (2008) noted that the TBQ has good reliability, a finding not replicated in Schachter et al. (2016) who found low reliability for this scale. It should also be noted that the TBQ does not contain items focused on children’s composing skills (assessing primarily teachers’ beliefs about transcription skills and how they should be supported), which may also have contributed to a lack of association. Challenges with both approaches for eliciting teachers’ beliefs and ideas suggests the need to more closely examine how researchers conceptualize and elicit teachers’ understanding of early writing. Given the complexity of early writing as a construct and the fact that teachers varied so much in their endorsement of the components within this construct, it is important that additional research be undertaken. This research should more carefully attend a full framework of early writing (e.g., Kaderavek et al., 2009; Puranik and Lonigan, 2014; Rowe and Wilson, 2015; Kim, 2020) and to teacher knowledge specifically because knowledge is an important source of teachers’ beliefs as noted by Hindman and Wasik (2008) and others (e.g., Leatham, 2006). But also, beliefs are central to teachers’ knowledge (Op ’t Eynde et al., 2002).

Although the method used in this study, inviting teachers to generate responses to an open-ended question, may have provided space for teachers to share all of their ideas about writing, it may have also limited responses. While this approach permitted teachers to openly share writing definitions, it is possible that the format proved challenging for teachers or may have generated less complex responses given the open nature of the prompt. Recent research by Bingham et al. (2022) suggests that teachers demonstrate extensive knowledge of writing, writing development, and supports for writing when asked to respond to children’s writing samples contextualized within a play experience, an approach that reflects typical practice of teachers. While this method elicited more details and depth of teachers’ knowledge than the isolated definition question used in this study, teachers’ responses to the contextualized writing samples were also narrow in focus, primarily targeting handwriting, motor skills, and print concepts, while their responses about composing and spelling were often inaccurate or vague (Bingham et al., 2022). This is an important finding with implications for the design of future elicitation materials for assessing beliefs and knowledge. Alternatively, there are benefits to belief measures that provide categories of responses for teachers like that of the TBQ. However, findings from this study suggest the need for an extended set of items that (a) reflect both the ideas of researchers and teachers and (b) comprehensively address research-based conceptualizations of early writing development.

### Beliefs and knowledge are related to specific practices

We found partial support for our hypothesis that teachers’ beliefs and definitions would be related to their classroom practices. One reason that we did not find additional associations may be related to the fact that previous research documents that preschool teachers enact few writing-related practices. In their study of teachers’ literacy beliefs, knowledge, and practice, Schachter et al. (2016) did not pursue attempts to link writing beliefs and knowledge with practices because, so few teachers were observed engaging young children in writing interactions. It is important to also acknowledge that previous research also documents challenges with linking early childhood teachers’ beliefs generally with their instructional practices (Hamre et al., 2012; Sandvik et al., 2014), particularly when examining reported beliefs and observed practices (McMullen et al., 2006; Schachter et al., 2016). Teachers may espouse to believe certain things, even strongly, but they may not engage daily in instructional practices to support these skills. This may be one reason that Schachter et al. (2016) found few or even negative associations between teachers’ early literacy beliefs and practices. Despite limited research has examined preschool teachers’ early writing beliefs and practices, others have noted limited concordance between teachers’ beliefs, what they say they do in their classrooms, and observed practices (Gerde et al., 2019b).

Although we didn’t find beliefs related to a wide range of early writing practices, early childhood teachers in this study who had less developmentally appropriate views of early writing or who demonstrated more limited understanding of writing development in their written responses were less likely to enact writing practices designed to support children’s early writing skills. For example, teachers’ beliefs as assessed by the TBQ were related to the number of teacher-child writing spelling interactions. That is, teachers who endorsed TBQ ideas were more likely to be observed supporting children’s writing by drawing attention to letter-sound correspondence and encouraging children to listen to the sounds in spoken language when attempting to write words they wanted to communicate with others. This association may be the result of the TBQ asking teachers to respond explicitly to ideas related to the need to be sensitive to young children as they build their orthographic knowledge and accepting emergent spelling attempts as developmentally appropriate rather than requiring precision in early spelling attempts. Although children’s invented or estimated spelling skills are just emerging in the preschool years (Puranik and Lonigan, 2011; Zhang et al., 2017), the ability to use letters and sounds to encode words is important to later writing and reading development (National Early Literacy Panel [NELP], 2008; Ouellette and Sénéchal, 2017). Hence, supporting teachers’ beliefs and understanding about how children develop early spelling skills, and how these can be supported in preschool classrooms, is likely a productive area of focus for early writing professional learning approaches. Importantly, researchers have offered guidance in how this instruction can be carried out in preschool classrooms in a manner that is developmentally appropriate for preschool aged children (Quinn et al., 2016; Copp et al., 2023).

We also found that teachers who have a more complex and thus, more complete understanding of the multiple components of early writing also provided more writing supports for children’s conceptual knowledge, a key feature of early writing. Previous research demonstrates that children in classrooms where teachers provide more supports for conceptual knowledge related to the purposes of writing, have higher invented spelling skills at the end of the year (Bingham et al., 2017). Teachers who understand that writing involves multiple writing components appear to be engaging in more practices to help children connect oral and written language as they engage in writing. Because writing concept or procedural knowledge is foundational to other early writing skills (Puranik and Lonigan, 2014), these types of instructional supports may be particularly helpful for young children in their development of both universal (how their ideas can be linked to written text) and language specific (how certain rules govern English writing) writing knowledge (Puranik and Lonigan, 2011; Treiman and Kessler, 2014). Notably, this finding expands previous work showing that teachers with a more complex knowledge of writing provide higher quality writing supports (Bingham et al., 2022) by pointing to a specific and meaningful component area–writing concept or conceptual knowledge–that is important for children’s writing development (Bingham et al., 2017). Given that there was wide variability among teachers in their writing concept focused interactions with children, teachers may benefit from professional learning approaches designed to support their beliefs, knowledge, and practices of this important writing skill.

### Limitations and future directions

A number of study limitations are important to acknowledge and have implications for areas of future research. First, we note that data presented in this study are correlational in nature and were collected at one point in time within the first few months of the beginning of school. The correlational nature means we cannot draw causal conclusions but also that the unidirectional relation between writing beliefs and practices is not well established. As teachers’ beliefs may develop or be influenced by their experiences across the school year, possibly in relation to the skill levels of children in their current classroom, future research is needed to examine how teachers’ beliefs and practices are related across the preschool year. As our data were collected in the fall of the school year, it is possible that once children had more experiences with writing in preschool settings that teachers’ beliefs and knowledge may have been slightly different. Additional assessment timespoints across the preschool year would answer critical questions related to how teachers’ beliefs and practices relate across time. Second, although we used qualitative and quantitative approaches for eliciting teachers’ beliefs and ideas about early writing development, we may have only partially captured these constructs. Because writing represents a number of distinct skills in early childhood, additional research is needed into approaches for holistically capturing teachers’ beliefs. This additional research should use multiple approaches for eliciting beliefs and examine how beliefs, self-efficacy beliefs, and knowledge relate to each other and practices across time. Alternative elicitation approaches (such as an interview) should also be explored as teachers may not have shared all their ideas about children’s writing development given the written format of the survey responses. Similarly, additional development is needed into survey-based approaches for eliciting writing beliefs with greater attention to writing components beyond handwriting and spelling. Of particular interest is how to support teachers’ knowledge and beliefs of writing that are developmentally appropriate in nature.

## Conclusion

We used quantitative and qualitative approaches to examine how teachers’ beliefs and ideas relate to their instructional practices. Findings suggest some variability in early childhood teachers’ writing beliefs; their definitions of writing heavily focused on handwriting skills. Results of this study have implications for the importance of teachers’ beliefs in supporting instructional practice, but also raise questions around the measurement of beliefs and knowledge. Teachers’ definitions of writing and their survey-based beliefs were unrelated to each other, but differentially related to the instructional focus of interactions with children. However, neither approach was related to the frequency of observed modeling or scaffolding behaviors. The heavy emphasis on handwriting skills in both teachers’ writing definitions and observed instructional practices suggests that in-service teachers possess a good understanding of children’s handwriting skills, but could use additional professional learning experiences designed to support their understanding of composing and early spelling skills, as well as how to support these in classroom practice. Because the knowledge base of early childhood teachers’ beliefs is still evolving, additional research into approaches for eliciting beliefs in comprehensive ways that is tied to instructional practice are clearly needed.

## Data availability statement

The original contributions presented in the study are included in the article/supplementary material, further inquiries can be directed to the corresponding author.

## Ethics statement

The studies involving humans were approved by the Georgia State University, Atlanta, Georgia, United States. The studies were conducted in accordance with the local legislation and institutional requirements. The participants provided their written informed consent to participate in this study.

## Author contributions

GB took the lead on data analysis and writing. Both authors helped to co-conceptualize this manuscript that was undertaken with data gathered from a shared research project.

## Footnotes

1
https://www.dedoose.com
